# 

**Published:** 1888-12-29

**Authors:** 


**Notice to Advertisers and Subscribers.**

All Advertisements, Orders for Copies of THE HOSPITAL, and Business Communications should be addressed to The Publisher, *not to the Editor*, THE HOSPITAL, 38, Old Jewry, E.C.

### AMUSEMENTS AND RELAXATION.

### NEW PUZZLE PRIZE SCHEME.

COMMENCED OCTOBER 6TH, ENDS DECEMBER 29TH, 1888.

On Oct. the 6th a New Prize Scheme was commenced in these columns.

Prizes will be awarded for the best Original Puzzles in Prose or Verse, relating to any of the *Social, Philanthropic, or Political Topics of the day*, and taking the form of Double Acrostics, Anagrams, Square Words, Decapitations, Transpositions, or any other Puzzles which the ingenuity of the competitors may suggest. (*Answers must accompany contributions.*)

*Readers* are requested to send in weekly the Number of Marks they consider each composition to be entitled to, three being the highest score for any one contribution.

Eight Prizes of Standard Books will be given, the choice of the book to be left to the winner.

First, Second, Third, Fourth, and Fifth Prizes for best Original Puzzles £1 1s., 15*s.*, 10 . 6*d.*, 7*s.* 6*d.*, and 5*s.* each.

First, Second, and Third Prize for Solutions of 15*s.*, 10*s.* 6*d.*, and 5*s.* each.

N.B.—All contributions must reach the office, 38, Old Jewry, E.C., not later than the *First Post* on *Thursday* in each week.

*No Competitor will be allowed to take more than one prize during a quarter.*

### PUZZLES FOR SOLUTION.

**Eleventh Week.**

No. 29. DOUBLE ACROSTIC.

May my primals to you bring
Hearty finals on their wing;
And may they both around us fling
Their brightest joys in everything.

1. A human need; by some I'm deemed
Of life the aim and end;
By others very little esteemed;
To all I prove a friend.

2. Some fearful sight of pain or woe,
Will fill you with my gloom;
And when through sorrow's paths you go,
My shadows round you loom.

3. " Oh! would that I in former days,
Had acted otherwise!"
In this and many other ways,
My hapless victim cries.

4. In the labour and toil of this bustling life,
There's many a chance and slip;
Lest the poor should sink in the weary strife,
I offer my friendly grip.

5. I should be submissive, obedient and mild,
Should honour authority too;
But I sometimes rebel, like a tiresome child,
And seek my own way to pursue.

6. Swift-footed, as all of my kind;
Timidly fearful of man;
I live on the praries, and find
The quiet I need by this plan.

7. I rise beside the poor man's cot,
And fill his babes with fear;
My inmates may relieve his lot,
And all his sorrows cheer.

8. When summer suns blaze overhead,
And shady spots are rare;
You'll have my shelter o'er you spread,
And seek refreshment there.

9. A quality none can despise;
Nor yet can prize too high;
Without it man can never rise,
Incapable he'll die.

PATIENCE.

No. 30. ORIGINAL ANAGRAMS.

1. Oh! Time escapes; no months left.
2. Hath not all pain use?

TINIE.

**Marks.**

| Names. | No. of Puzzles sent in Dec. 20. | No. of Marks Dec. 20. | Total of Marks. | Names. | No. of Puzzles sent in Dec. 20. | No. of Marks Dec. 20. | Total of Marks. |
|---|---|---|---|---|---|---|---|
| Patience...... | 3 | 7 | 58 | Hercules...... | — | — | 11 |
| Lady Clara... | 3 | 7 | 65 | Gretta......... | 3 | 7 | 53 |
| Tinie ......... | 3 | 8 | 56 | Dandy Dick | — | — | 5 |
| Bijou ......... | 2 | 3 | 31 | Boadicea ... | — | — | 7 |
| Thanet ...... | — | — | 30 | Nipper......... | 3 | 5 | 49 |
| G. D. ......... | — | — | 15 | Morico......... | 3 | 7 | 55 |
| Daisy ......... | 3 | 4 | 23 | A. M. E....... | — | — | 3 |
| Salford ...... | — | — | 6 | John Gilpin | — | — | 4 |

"**Erratum.**"—Read for "Gretta" total marks last week 46, not 42 as printed.

**Answers to Ninth Week Puzzles.**

No. 23. DOUBLE ACROSTIC.—

H ira M

O lympi A

S lu G

P anam A

I ne Z

T ahit I

A me N

L ouis E

No. 24. CONUNDRUM.—Because it is always a-musing (amusing).

No. 25. HOUR-GLASS PUZZLE.—
D e c e m b e R
E q u a t E
C h u B
E M
C l u B
E s c a p E
D e c e m b e R

**Marks for Solution of Puzzles.**

Total Marks.—Ruffles (1), 12; Daisy, (0), 7; Lady Clara (2), 12; Reldas (0), 9; Mater (0), 6; Condorcet, 7; Tinie (0), 11; Gentian (1), 14; Marguerite, 5; Patience, 7; Gretta, 4; Amicus, 7; Morico, 3; Scrooge (1), 13; Smut, 2; Thanet 15; Jenny Wren (0) 3; Lauriston, 1.

[For Conditions, see THE HOSPITAL for December 15th.]

*Second Quarterly Competition commences January 5th,* 1889.

